# Targeting Histone Deacetylases: A Novel Approach in Parkinson's Disease

**DOI:** 10.1155/2015/303294

**Published:** 2015-01-28

**Authors:** Sorabh Sharma, Rajeev Taliyan

**Affiliations:** Neuropharmacology Division, Department of Pharmacy, Birla Institute of Technology and Science, Pilani, Rajasthan 333031, India

## Abstract

The worldwide prevalence of movement disorders is increasing day by day. Parkinson's disease (PD) is the most common movement disorder. In general, the clinical manifestations of PD result from dysfunction of the basal ganglia. Although the exact underlying mechanisms leading to neural cell death in this disease remains unknown, the genetic causes are often established. Indeed, it is becoming increasingly evident that chromatin acetylation status can be impaired during the neurological disease conditions. The acetylation and deacetylation of histone proteins are carried out by opposing actions of histone acetyltransferases (HATs) and histone deacetylases (HDACs), respectively. In the recent past, studies with HDAC inhibitors result in beneficial effects in both *in vivo* and *in vitro* models of PD. Various clinical trials have also been initiated to investigate the possible therapeutic potential of HDAC inhibitors in patients suffering from PD. The possible mechanisms assigned for these neuroprotective actions of HDAC inhibitors involve transcriptional activation of neuronal survival genes and maintenance of histone acetylation homeostasis, both of which have been shown to be dysregulated in PD. In this review, the authors have discussed the putative role of HDAC inhibitors in PD and associated abnormalities and suggest new directions for future research in PD.

## 1. Introduction

Movement disorders are a group of nervous system disorders that primarily affect the basal ganglia and result in abnormal voluntary or involuntary movements. They are generally categorized as a group of neurological symptoms, signs, or diseases that manifest as either slowness or paucity of movement (hypokinesias; typically seen in Parkinson's disease (PD) and other parkinsonian disorders) or by excessive, abnormal involuntary movements (hyperkinesias) typically seen in Huntington's disease (HD), tremors, dystonia hemi-facial spasm, and akathisia [1, 2].

Among all the movement disorders, PD affects approximately 2% of the population over the age of 65 and is characterized by behavioral motor deficits including tremor, rigidity, bradykinesia, and postural instability. Selective dopaminergic neuronal degeneration in substantia nigra pars compacta (SNpc) is the prominent feature of PD pathology [2, 3]. Levodopa (L-DOPA) is a substitutive pharmacological agent that restores the physiological concentration of dopamine in the brain, particularly in the striatum. However, long term use of L-DOPA results in complications such as movement behaviour fluctuations and dyskinesia in most patients with PD. Thus, there is an unmet medical need to identify and explore new therapeutic options for PD, which should be effective and safe.

The past decade has produced an exponential increase in research examining the genetic and environmental factors that contribute to the etiology of PD [4]. The gene expression regulated by the chromatin, a densely packed complex structure containing DNA and histone proteins, has been found to be altered in patients of PD. There are five major families of histones: H1/H5, H2A, H2B, H3, and H4. Histones H2A, H2B, H3, and H4 are known as the core histones, while histones H1 and H5 are known as the linker histones [5]. These core histone proteins are subjected to posttranslational modifications on their N-terminal tails. These posttranslational modifications of histones are often dynamic and reversible and are mediated by two antagonistic sets of enzymatic complexes that attach or remove the corresponding chemical groups in a site-specific manner. Various posttranslational modifications include acetylation, phosphorylation, ubiquitination, SUMOylation, and ADP-ribosylation, all of which are capable of influencing the rate of gene transcription. One of the most thoroughly studied modification of histone tails is the acetylation at lysine residues [5].

Recent investigations suggest that gene expression modulated by histone acetylation might be associated with neurodegenerative processes [6, 7]. Histone deacetylases (HDACs) along with histone acetyltransferases (HATs) are the enzymes that regulate the homeostasis of histone acetylation. Inhibitors of HDACs, which were initially characterized as anticancer drugs, are recently suggested to act as neuroprotective agents that act by enhancing synaptic plasticity, neuronal survival, learning, and memory in a wide range of neurodegenerative disorders, including Alzheimer's disease (AD), PD, and HD [6–10]. Moreover, we have explored the neuroprotective role of HDAC inhibitors in high fat diet induced cognitive deficits in mice and also in intracerebroventricular streptozotocin induced AD in rats [11, 12]. In this review we have discussed the putative role of HDACs in PD and the potential of specific HDAC inhibitors as new pharmacological strategies for the treatment of PD.

## 2. Mechanism of Histone Acetylation

**Figure 1:**
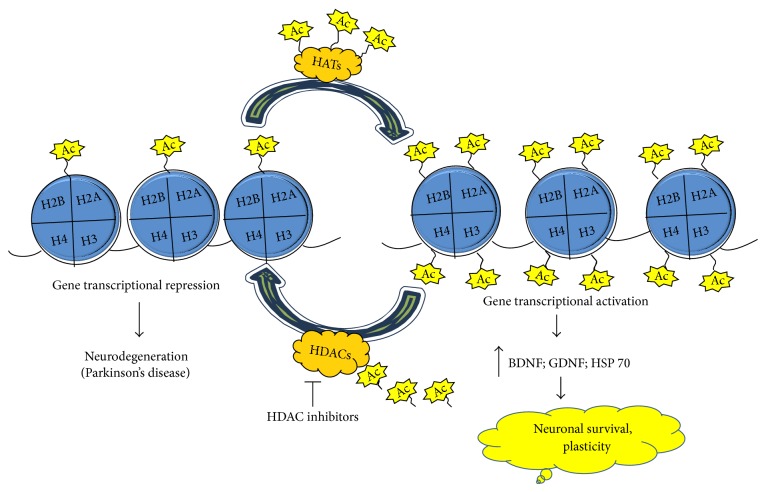
Transcriptional regulation by histone acetyltransferase and histone deacetylases. HAT: histone acetyltransferase, HDAC: histone deacetylases; HSP 70: heat shock protein 70, BDNF: brain derived neurotropic factor, GDNF: glial cell derived neurotropic factor, and PD: Parkinson's disease.

Histone acetylation is a chromatin modification that modulates histone-DNA interactions via two different classes of enzymes: HATs and HDACs (Figure 1). HATs are the enzymes that acetylate the lysine residues on N-terminal tails of core histones using acetyl-coenzyme A as an acetyl group donor. The addition of acetyl group neutralises the positive charge of the lysine residue, thus reducing the electrostatic interaction between the lysine in the histone tail and the negatively charged phosphate group on DNA. This causes a relaxation of chromatin, known as euchromatin and thus turns on the gene transcription, whereas on the other hand, deacetylation carried out by HDACs results in removal of the acetyl groups from lysine in the histone tail and thus restoring the positive charge and causing a condensation of chromatin, known as heterochromatin, thus turning off the gene transcription [13]. However, it is important to remember that it cannot be generalized that an increase in acetylation will induce an increase in transcription.

**Figure 2:**
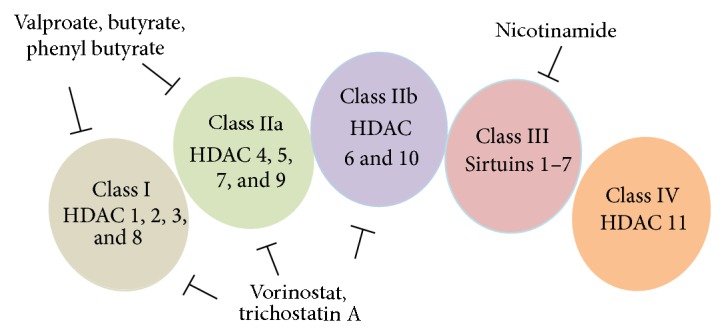
Classification of histone deacetylase families. HDACs: histone deacetylases.

In mammals the HDACs are divided into 4 classes based on their function and structural homologies to yeast HDACs. The classification of HDACs along with their pan inhibitors is provided in Figure 2 [14–16]. HDACs modulate both histone and nonhistone proteins. The nonhistone protein targets are transcription factors (e.g., p53, STAT1, or STAT3), cytoskeleton proteins (e.g., *α*-tubulin), and other cellular proteins (e.g., heat shock protein 90 (HSP 90) or KU70). Till date, more than 50 nonhistone proteins have been identified as substrates for HDACs. The structure and activity of these nonhistone proteins may be altered by acetylation/deacetylation with consequent effects on various cell functions including gene expression, cell cycle progression, and cell death pathways. Classes I, II, and III of HDACs regulate lysine acetylation of these nonhistone proteins that exert neuroprotective effects [17, 18] adding some complexity to the interpretation of therapeutic potentials of currently available broad spectrum or even class specific HDAC inhibitors for neurodegenerative diseases. For detailed study of these nonhistone targets refer to [19, 20]. HDACs are expressed in multiple tissues, including the brain and spinal cord. Although some HDAC family members have limited tissue specificity and central nervous system (CNS) distribution, individual neurons frequently express multiple HDAC (Table 1) [21–34]. Rouaux and colleagues were the first to identify alterations of histone acetylation levels in neurodegeneration, by demonstrating that histone acetylation levels were decreased globally in neurons [35]. Since then, the linkage between histone hypoacetylation and neurodegeneration has been well established in numerous cognitive and movement disorders, including AD, PD, and HD [36, 37].

## 3. Historical Aspects of HDACs and HDAC Inhibitors

**Figure 3:**
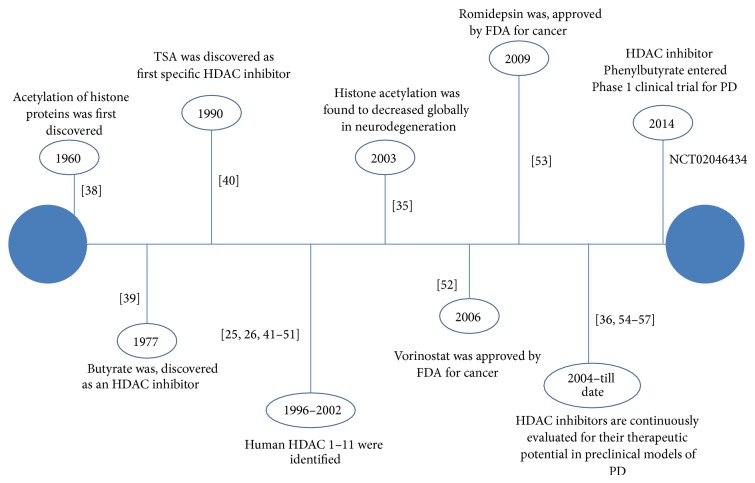
Historical aspects of HDACs and their modulators.

The isolation, purification, and identification processes of various HDAC isoforms begin right after the discovery of histone acetylation phenomenon. Thereafter, various HDAC inhibitors have been synthesized and studied which helps to explore the pharmacological actions of HDACs. A timeline figure regarding the historical aspects of HDACs has been provided (Figure 3) [25, 26, 38–57].

## 4. HDAC Inhibitors and Parkinson's Disease

### 4.1. HDAC Inhibitors in Animal Models of Parkinson's Disease

#### 4.1.1. MPTP (1-Methyl-4-phenyl-1,2,3,6-tetrahydropyridine)

Initially, MPTP was identified as a strong neurotoxin when heroin addicts accidentally self-administered MPTP and developed an acute form of Parkinsonism that was indistinguishable from idiopathic PD [58]. MPTP has been widely used as an animal model to replicate human PD symptoms in nonhuman primates and in rodents. MPTP is a highly lipophilic agent that following systemic injection (usually i.p or s.c) rapidly crosses the blood-brain barrier. Once inside the brain, MPTP is converted by MAO-B (monoamine oxidase) into a neurotoxin precursor, MPP+ (1-methyl-4-phenylpyridinium), which causes selective destruction of dopaminergic neurons in the SNpc (substantia nigra pars compacta). MPP^+^ induces neurotoxicity primarily by inhibiting complex I of the mitochondrial electron transport chain, resulting in ATP depletion and increased oxidative stress [3, 59]. The therapeutic potential of HDAC inhibitors has been evaluated in MPTP induced neuronal toxicity in rodent models. Generally, HDAC inhibition in the brain has been associated with the increased transcription of free radical scavengers, heat-shock proteins, and antiapoptotic bcl-2 family members that may contribute to the protection of dopaminergic neurons following MPTP exposure [60].

Phenylbutyrate was among the very first HDAC inhibitors to be tested in MPTP model. Pretreatment of MPTP intoxicated mice with phenylbutyrate significantly attenuated the loss of dopamine and its metabolites 3,4-dihydroxyphenylacetic acid (DOPAC) and homovanillic acid (HVA) in the striatum. Moreover, phenylbutyrate also protects tyrosine hydroxylase (TH^+^) neurons from MPTP induced toxicity, which is basically a rate limiting enzyme in dopamine biosynthesis [54].

Neuroinflammation and oxidative stress have been well reported to play a key role in the pathophysiology of PD [61, 62]. Increased levels of inflammatory and oxidative stress markers along with decreased endogenous antioxidants level have been observed in both animal models and PD patients [59–63]. Recently, oral administration of sodium phenylbutyrate in MPTP treated mice has been reported to suppress the expression of proinflammatory markers, nuclear factor-kB (NF-kB), and reactive oxygen species in activated glial cells. Activation of small G proteins p21^ras^ and p21^rac^ has been linked with generation of neuroinflammation and oxidative stress after MPTP intoxication. The authors suggested that phenylbutyrate results in reduced nigral activation of p21^ras^ and p21^rac^ G proteins and hence results in dopaminergic neuronal protection along with improved motor functions in MPTP-intoxicated mice [64]. Along with oxidative stress and neuroinflammation, excitotoxicity has also been reported to play pathogenic role in development of PD [65]. Glutamate, a well-known excitatory amino acid has been found to be elevated in PD patients [65]. The therapeutic potential of HDAC inhibitors has been evaluated against glutamate induced toxicity and it was reported that TSA, a HDAC inhibitor, prevents glutamate induced toxicity in the medium of MPP^+^ treated primary cultured astrocytes [66]. The authors suggested that TSA alleviates MPP+-induced impairment of astrocytic glutamate uptake, which might be a novel mechanism contributing to neuroprotection by HDAC inhibitors.

Olfactory and cognitive deficits have been reported very frequently in PD. A study shows that valproate pretreatment in rats infused with a single intranasal administration of MPTP was able to prevent olfactory discrimination and short-term memory impairments as evaluated in the social recognition and step-down inhibitory avoidance tasks. Moreover, valproate alone or in combination with lithium prevented dopamine depletion in the olfactory bulb and striatum of MPTP-infused rats [59]. In another study, valproate pretreatment has been found to protect midbrain dopaminergic neurons from MPP^+^-induced neurotoxicity [67] and upregulate the expression of neurotrophic factors, including glial cell line-derived neurotrophic factor (GDNF), and brain derived neurotrophic factor (BDNF) from astrocytes. The expression of these neurotrophins has been reported to be decreased in animal models as well as in postmortem brains of PD patients [68, 69]. Other HDAC inhibitors such as vorinostat, TSA, and sodium butyrate have also been reported to mimic the neuroprotective and survival promoting effects of valproate on dopaminergic neurons in neuron-glia cultures [56, 70, 71]. All these HDAC inhibitors were demonstrated to increase the expression of GDNF and BDNF in astrocytes along with marked increase in histone H3 acetylation. Taken together, these studies highlight that HDAC inhibitors upregulate GDNF and BDNF expression in astrocytes and protect dopaminergic neurons, at least in part, through HDAC inhibition and consequential H3 acetylation.

As discussed earlier, MPP^+^ the toxic metabolite of MPTP acts by selectively inhibiting complex I of mitochondria. In a recent study, mitochondrial fragmentation was found to be an early event during apoptosis caused by MPP+ in SH-SY5Y cells. TSA selectively rescues mitochondrial fragmentation and cell death induced by lower doses of MPP^+^. The mitochondrial fragmentation occurring as a result of MPP^+^ treatment could possibly be mediated through downregulation of Mfn2. However, TSA administration results in complete reversal of Mfn2 expression. Further investigation suggests that TSA prevents MPP^+^-induced Mfn2 downregulation via inhibiting HDAC over Mfn2 promoter and alleviating its transcriptional dysfunction [72]. This study implicates that mitochondrial fragmentation, an early event during neuronal apoptosis in PD, may be a result of alteration in the acetylase/deacetylase balance and also indicates that HDAC inhibitors might be a potential early treatment for PD.

In their later study, Kidd and Schneider found the neuroprotective effects of valproate in MPTP intoxicated FVBn mice. Valproate partially prevents striatal dopamine depletion and almost completely protects dopaminergic cell loss in SNpc. These neuroprotective effects of valproate were attributed to its HDAC activity as increased acetylation of histone 3 lysine 9 was observed in SNpc of FVBn mice [73]. In another study, the therapeutic potential of valproate, sodium butyrate, and vorinostat was tested against MPP^+^-mediated cytotoxicity in human derived SK-N-SH and rat derived MES 23.5 cells. These HDAC inhibitors partially prevented MPP^+^-mediated apoptotic cell death. The protective effects of these drugs coincided with significant increases in histone acetylation [74].

The majority of PD cases are sporadic; that is, only about 10% of patients report a positive family history [2, 75]. Out of the various genes unequivocally linked to monogenic PD, mutations in PARK 2, 6, and 7 are responsible for autosomal-recessive PD. The DJ-1 protein has been repeatedly associated with early-onset, autosomal recessive Parkinson disease (PARK7). As low levels of this protein increase the risk for PD [30], it is obvious to find a therapeutic strategy which could results in increased expression of DJ-1. Recently, the HDAC inhibitor, phenylbutyrate, has been demonstrated to increase DJ-1 expression by 300% in the N27 dopamine cell line and rescues cells from oxidative stress and mutant alpha-synuclein (*α*-syn) toxicity. *α*-syn forms abnormal protein deposits in dopamine neurons and is believed to cause the death of brain cells, leading to PD. Moreover, in mice intoxicated with MPTP, phenylbutyrate treatment results in protection of dopaminergic neurons as well as increasing DJ-1 levels in brain. In addition, long-term administration of phenylbutyrate reduces *α*-syn aggregation in brain and prevents age-related deterioration in motor and cognitive function in a transgenic mouse model of diffuse Lewy body disease [30].

The major complication in PD treatment with chronic L-DOPA is the occurrence of L-DOPA induced dyskinesias (LIDs). Various therapeutic strategies have been adopted to delay the use of L-DOPA as much as possible or by finding a suitable treatment option to reduce the dose of L-DOPA. However, none of these options fully serves the purpose and rendering LIDs, a major hurdle in PD treatment. However, recently, HDAC inhibitor, RGFP109, has been demonstrated to attenuate LIDs in the MPTP-lesioned marmoset [76]. RGFP109 has been well reported to penetrate into brain and have an oral bioavailability of 35% as reported in dogs [76]. Importantly, the authors demonstrate that antidyskinetic effects of RGFP109 were obtained without compromising peak antiparkinsonian efficacy or duration of L- benefit, which suggests that abnormal HDAC activity is a consequence of LID and not l-DOPA therapy* per se*.

#### 4.1.2. 6-Orthohydroxydopamine (6-OHDA)

The neurotoxin 6-hydroxydopamine (6-OHDA) continues to constitute a valuable tool used in modelling PD in rodents. To target specific neurons and to bypass the blood-brain barrier, 6-OHDA is typically injected stereotactically into the brain region of interest. The classical method of intracerebral infusion of 6-OHDA involves a massive destruction of nigrostriatal dopaminergic neurons. Once it enters the brain, 6-OHDA accumulates in the cytosol where it is readily oxidized leading to the generation of reactive oxygen species and ultimately oxidative stress-related cytotoxicity. To date, 6-OHDA is widely used to lesion the nigrostriatal dopaminergic system as a model of PD [77–79]. Multiple intrastriatal injections of this neurotoxin result in rapid loss of dopaminergic terminals in the striatum itself followed by slower (3-4 weeks) and partial retrograde degeneration of dopaminergic neurons in the SNpc [77, 78]. 6-OHDA administration into the striatum leads to shrinkage of this area along with loss of TH^+^ neurons in rats. The HDAC inhibitor, valproate, has been reported to attenuate 6-OHDA induced toxicity in rats and results in sparing of TH^+^-immunoreactivity and elevation of TH^+^ content in SNpc as well as striatum. Also, valproate treatment increased the expression of *α*-syn in SNpc and striatum as compared to 6-OHDA treated rats [78]. These results are in agreement with the previous study of same authors in which they confirmed the prosurvival role of *α*-syn in this model [77]. This prosurvival role of *α*-syn seems awkward and is still debateable. While its aggregation is usually considered linked to neuropathology of PD, its normal function may be related to fundamental processes of synaptic transmission and plasticity. The authors demonstrate that *α*-syn silencing in cultured cerebellar granule cells results in widespread death of these neurons. In contrast, treatment with HDAC inhibitor, valproate, results in increased expression of *α*-syn and preventing its monoubiquitination and nuclear translocation in cerebellar granule cells exposed to 6-OHDA [77].

As mentioned earlier, PD patients often experience cognitive impairment during the disease progression. Recently, Rane and colleagues studied the effect of sodium butyrate in 6-OHDA induced cognitive deficit in premotor stage of PD and they found sodium butyrate to be highly effective in attenuating cognitive deficits in 6-OHDA administered rats [79]. We have also demonstrated the neuroprotective effects of HDAC inhibitors, vorinostat, and TSA in 6-OHDA induced hemiparkinsonism in rats in our laboratory and we found that HDAC inhibition results in improved motor activity as assessed by narrow beam walk and wire suspension tasks. Moreover, the rats treated with HDAC inhibitors results in significant increase in histone H3 acetylation as compared with 6-OHDA treated rats (unpublished data). Thus, these reports suggest that HDAC inhibition could be of therapeutic importance in restoring motor and cognitive disabilities associated with PD.

#### 4.1.3. Rotenone

Rotenone is a strong inhibitor of mitochondrial complex I, which is located at the inner mitochondrial membrane and protrudes into the matrix. It has been demonstrated that the chronic systemic exposure to rotenone develops many features of PD, including nigrostriatal dopaminergic degeneration [80]. Rotenone administration in rats results in activation of various pathogenic pathways including oxidative stress, *α*-syn phosphorylation and aggregation and lewy body pathology and so forth [80] and also reproduces all the behavioral features seen in the typical form of human PD [62]. Recently, chronic administration of valproate significantly protects nigrostriatal dopaminergic neurons and counteracts the drop in striatal dopamine levels caused by rotenone administration in rats. Further, valproate treatment prevents alterations in *α*-syn and attenuates loss of TH^+^ in SNpc and striatum of rotenone treated rats [81]. Valproate treatment has also been reported to attenuate apoptosis in neuroblastoma cells, SHSY5Y, a human dopaminergic cell line often used for study of PD [82]. The authors revealed that neuroprotective effects of valproate occurred as a result of cytochrome-c (cyt-c) inhibition along with decreased production of caspase-9 and caspase-3. Valproate treatment also results in increased expression of HSP70 protein and this activation of HSP70 could be a consequence of HDAC inhibition [83]. Another HDAC inhibitor, Phenylbutyrate has been demonstrated to attenuate motor deficits and reduced *α*-Syn accumulation induced by repeated rotenone administration in C57BL/6 mice [84]. Recently, sodium butyrate has also been demonstrated to reduce degeneration of dopaminergic neurons in a mutant *α*-syn drosophila transgenic model of familial PD. Chronic exposure to rotenone causes locomotor impairment and early mortality in drosophila. However, treatment with 10 mM sodium butyrate-supplemented food rescued the rotenone-induced locomotor impairment and early mortality in flies [57]. In addition, it was found that flies with the genetic knockdown of HDAC activity were resistant to rotenone-induced locomotor impairment and early mortality. This is one of the very few studies which indicate the beneficial effect of HDAC gene knockout in animal models. Moreover, the authors showed increased dopamine levels in brains of flies after sodium butyrate treatment as compared to alone rotenone treated flies. Thus, the above studies highlight the importance of HDAC inhibitors in attenuating rotenone induced toxicity in animals and cell culture models and support the notion that HDAC inhibitors result in increased transcription of neuronal survival genes and hence provide neuroprotection [85].

#### 4.1.4. Lipopolysaccharide (LPS)

Neuroinflammation triggered by activated microglial cells results in deleterious events, that is, oxidative stress and cytokine-receptor-mediated apoptosis, which might eventually lead to dopaminergic cell death and PD progression [61]. Lipopolysaccharide (LPS), an endotoxin from Gram-negative bacteria, acts as a potent stimulator of microglia and has been used to study the inflammatory process in the pathogenesis of PD. Various studies have highlighted the neuroprotective role of HDAC inhibitors in LPS-treated neuroglia cultures through the inhibition of release of proinflammatory cytokines and chemokines from microglia [86, 87]. These authors suggest that this reduced neuroinflammation could be achieved as a result of microglial apoptosis and treatment with HDAC inhibitors, TSA and sodium butyrate and valproate, has been found to reduce the number of microglial cells [86]. In line with these studies, TSA administration has also been reported to reduce the expression of numerous cytokines and chemokines in primary human fetal microglial cultures activated by LPS [88]. Further, HDAC inhibitors have been demonstrated to increase neurite formation, interneuronal networks, and number of TH^+^ neurons in LPS-pretreated cultures along with increased expression of BDNF and GDNF from astrocytes [56, 70]. These reports suggest that, along with increased expression of neurotrophic factors, the HDAC inhibitors also have significant impact on inflammatory conditions. Based upon these results, it is highly conceivable that neuroinflammation, which is mostly mediated by activated microglial and peripheral immune cells, can be controlled with the use of HDAC inhibitors.

### 4.2. *α*-Synuclein Toxicity

As discussed above, *α*-syn is a neuronal protein implicated genetically in PD and whose exact role is still controversial. However, it has been demonstrated in drosophila model that a nuclear localization of *α*-syn promoted its toxicity through increased binding of *α*-syn to histones which ultimately results in reduced levels of acetylated histone H3, moderates HAT activity, and inhibits HAT-mediated acetyltransferase activity [36]. In contrast, treatment with HDAC inhibitors, sodium butyrate and vorinostat, in SH-SY5Y cells or transgenic flies, rescued *α*-syn-induced toxicity and decreased neuronal cell death, suggesting that HDAC inhibitors may represent a promising therapeutic strategy to mitigate the progressive neurodegeneration linked to PD [36]. In another study, it has been demonstrated that inhibition of SIRT2, a Class III HDAC, by a potent inhibitor results in reducing the *α*-syn toxicity and modified inclusion morphology in a cellular model of PD. Moreover, SIRT2 inhibitors result in neuroprotection against dopaminergic cell death in both* in vitro* and a drosophila model of PD [89]. From these studies, it can be concluded that increased levels of nuclear *α*-syn in PD lead to decreased histone acetylation and neurotoxicity. Thus, the treatments that raise the levels of neuronal acetylation (i.e., HDAC inhibitors) may be attractive therapeutic strategies in PD. However, most of these beneficial effects observed in animal studies were obtained from pretreatment with HDAC inhibitors.

### 4.3. HDAC Inhibitors in PD Patients and Clinical Trials

Although initial trials with valproate did not significantly alter Parkinson features in PD patients [90], however, in a double-blind crossover trial with 12 PD patients, sodium valproate administration at 1200 mg daily results in slight to moderate improvement in LIDs. Moreover, excess salivation was found to be improved in four subjects with sodium valproate treatment [91]. Recently, a nonrandomized Open Label Phase I clinical trial (NCT02046434) of the FDA approved drug phenylbutyrate was started to observe the therapeutic potential of this drug in enhancing the removal of *α*-syn from the brain into the bloodstream (ClinicalTrials.gov identifier: NCT02046434). However, as such no results in PD patients are still available.

## 5. Future Directions and Conclusions

**Figure 4:**
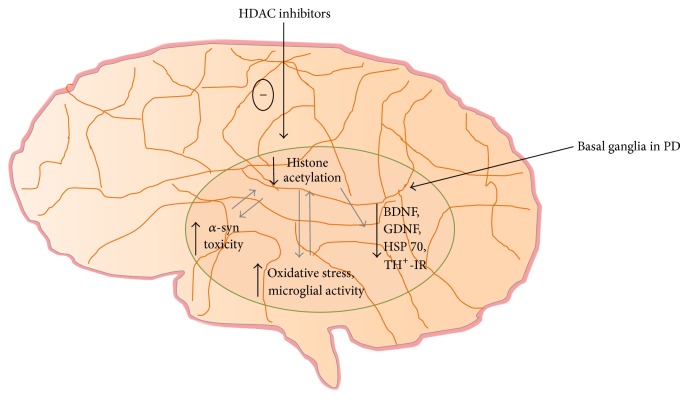
Neuroprotective mechanisms exerted by HDAC inhibitors. HSP 70: heat shock protein 70, BDNF: brain derived neurotropic factor, GDNF: glial cell derived neurotropic factor, *α*-syn: alpha-synuclein, and TH^+^-IR: tyrosine hydroxylase immunoreactivity.

HDAC inhibition is a validated approach in cancer therapy, as evidenced by the FDA approval of vorinostat and romidepsin followed by encouraging clinical data from other HDAC inhibitors. Recent evidences reveal that HDAC inhibitors could play an important role in various brain diseases. Chronic dysregulation of the acetylation/deacetylation activities can ultimately lead to neuronal cell death as manifested in neurodegenerative diseases. Thus, more research is required to fully understand the precise mechanism(s) by which this system impacts neuronal survival. This study has summarised and described the most prevalent movement disorder, that is, PD that involve alterations in histone modifications which can be reversed, at least in part, by treatment with HDAC inhibitors. HDAC inhibitors have specific effects on gene expression, upregulating a selective set of target genes and reducing expression of others. The neuroprotective effects of various HDAC inhibitors are accomplished either through increased histone acetylation or through the increased transcription of genes encoding neurotropic factors (BDNF, GDNF), HSPs or reduction in the accumulation of neurotoxic proteins (*α*-syn), and so forth (Figure 4). Although there are some reports indicating the beneficial effects of HDAC inhibitors in human subjects suffering from LIDs and some HDAC inhibitors have recently entered clinical trials for PD, still there are much awaited results. Moreover, some of the clinical studies have reported no improvement in PD symptoms when treated with HDAC inhibitors. It might be because of the multiple target approach of these HDAC inhibitors. Nevertheless, these data provide support for the continued study of the role of epigenetic modifications in PD and associated abnormalities and the potential of HDAC inhibition as a treatment for motor complications of in PD.

## Figures and Tables

**Table 1:** Histone deacetylases in brain.

HDAC class	Isoforms expressed in brain	Localization	Species	References
Class 1 (Zn^2+^ dependent)	HDAC 1	Cortex, caudate/putamen, hippocampus, amygdala SNpc, SNpr, locus coeruleus, corpus callosum, gray matter, white matter	Human, mouse, rat	[21–24]
	HDAC 2	Cortex, caudate/putamen hippocampus, amygdala SNpc, SNpr, locus coeruleus, gray matter, white matter, corpus callosum	Mouse, rat, mouse	[21–24]
	HDAC 3	Cortex, caudate/putamen hippocampus, amygdala SNpc, SNpr, locus coeruleus, globus pallidus	Mouse, rat, human	[21–23]
	HDAC 8	Hippocampus, amygdala SNpc, locus coeruleus	Human, rat	[21, 22, 25]

Class IIa (Zn^2+^ dependent)	HDAC 4	Cortex, caudate/putamen, hippocampus, amygdala SNpc, SNpr, locus coeruleus, globus pallidus	Human, mouse, rat	[21, 22, 24, 26, 27]
	HDAC 5	Cortex, caudate/putamen, hippocampus, amygdala SNpc, SNpr, locus coeruleus, globus pallidus	Rat, mouse, human	[21, 22, 28]
	HDAC 7	Cortex, caudate/putamen, hippocampus, amygdala SNpc, locus coeruleus, striatum, cerebellum	Rat, mouse, human	[21, 22, 29]
	HDAC 9	Cortex, SNpc, hippocampus, amygdala	Human, mouse, rat	[21, 22, 30, 31]

Class IIb (Zn^2+^ dependent)	HDAC 6	Cortex, caudate/putamen Hippocampus, amygdala, SNpc, locus coeruleus, cerebellum	Human, mouse, rat	[21, 22, 32]
	HDAC 10	Cortex, amygdala, hippocampus	Human, mouse, rat	[21, 22, 33]

Class IV (Zn^2+^ dependent)	HDAC11	Cortex, hippocampus, brain stem, cerebellum, diencephalon	Human, mouse, rat	[21, 22, 34]

SNpc: substantia nigra pars compacta, SNpr: substantia nigra pars reticulata, and HDAC: histone deacetylase.

## Abbreviations

AD: Alzheimer's disease
GSK 3*β*: Glycogen synthase kinase 3*β*
HDAC: Histone deacetylase
HAT: Histone acetyl transferases
HSP 70: Heat shock protein 70
HD: Huntington's disease
PD: Parkinson's disease.
